# Forecasting the length-of-stay of pediatric patients in hospitals: a scoping review

**DOI:** 10.1186/s12913-021-06912-4

**Published:** 2021-09-08

**Authors:** Natália B. Medeiros, Flavio S. Fogliatto, Miriam K. Rocha, Guilherme L. Tortorella

**Affiliations:** 1 Department of Industrial Engineering, Universidade Federal do Rio Grande do Sul, Av. Osvaldo Aranha, 99, 5° andar, Porto Alegre, 90035-190 Brazil; 2 Center of Engineering, Universidade Federal do Semi-Árido, Rua Francisco Mota Bairro, 572 - Pres. Costa e Silva, Mossoró, RN 59625-900 Brazil; 3 Department of Mechanical Engineering, The University of Melbourne, Melbourne, Australia; 4 IAE Business School, Universidad Austral, Buenos Aires, Argentina; 5 Department of Industrial Engineering, Universidade Federal de Santa Catarina, Campus Universitário Reitor João David Ferreira Lima, s/n°, Florianópolis, SC 88040-900 Brazil

**Keywords:** Hospital length-of-stay, Forecasting models, Pediatric patients, Neonatal patients, Scoping review

## Abstract

**Background:**

Healthcare management faces complex challenges in allocating hospital resources, and predicting patients’ length-of-stay (LOS) is critical in effectively managing those resources. This work aims to map approaches used to forecast the LOS of Pediatric Patients in Hospitals (LOS–P) and patients’ populations and environments used to develop the models.

**Methods:**

Using the Preferred Reporting Items for Systematic reviews and Meta-Analyses extension for Scoping Reviews (PRISMA-ScR) methodology, we performed a scoping review that identified 28 studies and analyzed them. The search was conducted on four databases (Science Direct, Scopus, Web of Science, and Medline). The identification of relevant studies was structured around three axes related to the research questions: (i) forecast models, (ii) hospital length-of-stay, and (iii) pediatric patients. Two authors carried out all stages to ensure the reliability of the review process. Articles that passed the initial screening had their data charted on a spreadsheet. Methods reported in the literature were classified according to the stage in which they are used in the modeling process: (*i*) pre-processing of data, (*ii*) variable selection, and (*iii*) cross-validation.

**Results:**

Forecasting models are most often applied to newborn patients and, consequently, in neonatal intensive care units. Regression analysis is the most widely used modeling approach; techniques associated with Machine Learning are still incipient and primarily used in emergency departments to model patients in specific situations.

**Conclusions:**

The studies’ main benefits include informing family members about the patient’s expected discharge date and enabling hospital resources’ allocation and planning. Main research gaps are associated with the lack of generalization of forecasting models and limited reported applicability in hospital management. This study also provides a practical guide to LOS–P forecasting methods and a future research agenda.

**Supplementary Information:**

The online version contains supplementary material available at 10.1186/s12913-021-06912-4.

## Background

With the increasing demand for health services, hospitals worldwide are operating under pressure to increase patient care quality while ensuring organizational survival [1]. The hospital environment is complex and imposes several managerial challenges related to optimal utilization of limited resources and the constant need to improve efficiency and reduce patients’ length-of-stay (LOS) [2]. Resource utilization planning requires predicting patients’ LOS since longer times imply lower turnover and higher costs, affecting the quality of patient care and reducing the availability of services to the population [3].

Forecasting pediatric patients’ LOS (LOS–P) in hospitals is particularly important since pediatric departments constantly struggle with capacity and overcrowding restrictions [2, 4], which could be avoided by predicting the use of hospital resources, and better dimensioning of care and hospitalization capacity [5]. In addition, forecasting LOS-P allows hospital managers to inform family members about the patient’s expected discharge date. We identified in the literature five dimensions positively affected by the use of forecasting models, which are presented in detail in the Discussion section; they are: (*i*) patient care, (*ii*) costs, (*iii*) hospital management, (*iv*) quality measurement, and (*v*) updating of medical practices.

Forecasting patients’ LOS is a subject widely studied in the literature through different methods and applications. Some studies use simple methods such as linear regression analysis (e.g., [6]), while more current studies use artificial intelligence techniques based on machine learning and deep learning (e.g., [7]). Literature review studies devoted to forecasting patients’ LOS in hospitals mainly analyze the adult population. Almashrafi et al. [8] and Peres et al. [9] conducted systematic literature reviews to find predictors of patients’ LOS in hospitals that should be considered in the generation of forecasting models. Atashi et al. [10], Hussain and Dunn [11], Lu et al. [12], and Verburg et al. [13] focused their reviews on the quality of the models proposed in the literature aiming at establishing a benchmark. Seaton et al. [14] was the only literature review that analyzed the pediatric population, identifying important factors when predicting neonates’ LOS in neonatal units.

In this article, we present a scoping review of the literature on LOS–P forecasting, addressing the current lack of studies that analyze the existing theoretical framework on the topic and map the main approaches used in the generation of LOS–P forecasting models. We present the main modeling techniques, their benefits and limitations, the environments investigated, and the types of pediatric populations considered. We close the article by presenting practical implications and directions for future research.

## Methods

The research method follows the methodology proposed by Arksey and O’Malley [15], which comprises five steps: (*i*) identification of research questions, (*ii*) identification of relevant studies, (*iii*) selection of studies, (*iv*) mapping of the data, and (*v*) collection, summarization, and reporting of the results. These steps are subsequently detailed. Scoping reviews provide a transparent and rigorous mapping of a research area, producing an accessible summary of the research results and indicating the existing gaps [15]. Scoping reviews are suitable for topics with scarcity of studies and whose body of knowledge has not been consolidated through a systematic literature review.

This research was driven by elements related to the forecasting of LOS–P, namely: techniques used, environments and populations in which techniques were applied, and results generated by the models created. Based on these elements, the study addressed five research questions:

- **RQ**_**1**_**.** What are the main techniques used to forecast LOS–P?
- **RQ**_**2**_**.** In which situations are those techniques applied?
- **RQ**_**3**_**.** What are the main characteristics of the data used to predict LOS–P?
- **RQ**_**4**_**.** What are the managerial implications of using LOS–P forecasting models for resource management in hospitals?
- **RQ**_**5**_**.** What are the main barriers and limitations of the existing studies and opportunities for future research?

The identification of relevant studies was structured around three axes related to the research questions: (*i*) forecast models, (*ii*) hospital length-of-stay, and (*iii*) pediatric patients. Those axes led to a combination of keywords used in the search (Table 1) and the research strategies used in each database in Supplementary Material 1. Four databases were consulted: Science Direct, Scopus, Web of Science, and Medline. The first three bases were chosen following the recommendation of Tortorella et al. [16]. The inclusion of Medline was due to its recurrent use in review articles on hospital LOS forecasting, e.g., [11, 13, 14]. The first axis should be present in the title, abstract, or keywords in our search, while the second and third axes should be present only in the title. The search was carried out between August 27 and September 11, 2020, using the CAPES platform (https://www.periodicos.capes.gov.br), which is a unified platform provided by the Brazilian Ministry of Education. We selected journal or conference articles published in English with no restriction with respect to publication date, resulting in a total of 821 articles. We did not consider (*i*) studies not yet been published or published as pre-prints due to the possibility that they may eventually not comply with the quality requirements of peer-reviewed journals and conferences, and (*ii*) conference abstracts and clinical tests, since they lacked sufficient information for the analysis.

**Table 1:** Research axes and keywords

*Research axes*	Forecast models	Length of stay in hospital	Pediatric patients
*Keywords*	Predict^a^	“Length of Stay”	Child^a^
	Model	“Hospital Days”	Pediatric
	Prognos^a^	“Length of Hospital Stay”	Paediatric
	Forecast^a^	“Duration of Stay”	Kid
	Regression	“Patient Stay”	Youth^a^
	Estimat^a^		Adolescen^a^
			Neonat^a^
			Newborn^a^
			Infant^a^

^a^is used so that the search returns all terms that begin with the word followed by the asterisk. Enclosing a sentence in double-quotes ensures that the search returns only documents in which the sentence appears and not just the words in any order

**Fig. 1:**
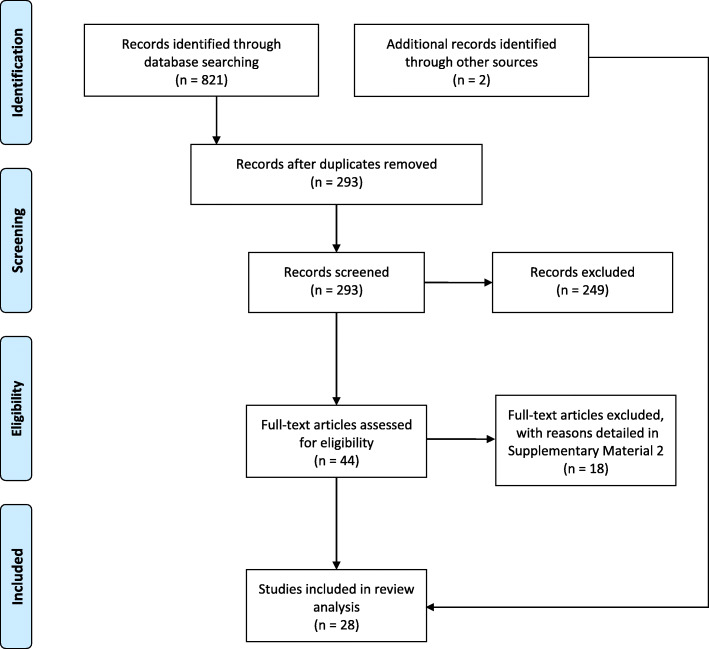
Results of PRISMA-Scr stages

To conduct the selection of studies, we followed the guidelines of Preferred Reporting Items for Systematic reviews and Meta-Analyses extension for Scoping Reviews (PRISMA-ScR )[17], as displayed in Fig. 1. The first two authors primarily carried out all stages to ensure the reliability of the review process. Differences in opinions were discussed with the remaining authors to reach a consensus. After this screening, data charting was done by the main author using an electronic spreadsheet; discrepancies discovered at this stage were addressed by all authors.

The identification stage was performed by searching for the axes in the chosen databases. The screening stage included two filters: (*i*) exclusion of duplicates and (*ii*) inclusion/exclusion criteria. The first filter excluded 528 duplicates; 293 publications remained. The remaining articles’ titles and abstracts were verified according to the second filter’s inclusion/exclusion criteria.

To be included in this review, studies (i.e., concept) should develop mathematical models to predict patients’ LOS in hospitals (i.e., context), and the studied population should be pediatric patients (i.e., population). Publications were excluded when the dependent variable was not the LOS in hospitals or investigated the effects of individual factors on LOS–P. After applying the inclusion/exclusion criteria, 44 articles remained. The eligibility stage consisted of a full-text analysis identifying articles aligned with the proposed research questions, resulting in 26 articles. In the final stage (inclusion), the selected articles’ references were checked to identify studies potentially aligned with the research theme and not identified during searches in the selected databases. Based on this backward snowballing technique, two articles were included totaling 28 publications in the final *corpus*. All articles were published in peer-reviewed journals and conferences, as informed on their webpages.

According to Arksey and O’Malley [15], data mapping must extract key information from the studies reviewed to help readers make decisions. We used the descriptive-analytical method to build an analytical structure, collecting standard information about each study. The following information was retrieved from our *corpus*, allowing the mapping of the literature: authors, year of publication, country of origin of the first author, journal or conference title, area of knowledge of the publication, application focus (i.e., department and population), data characteristics, techniques used to build the forecast model, limitations, contributions, future research directions (see Supplementary Material 3). We used the information extracted during the data mapping to group, summarize, and present the results in a logical and organized way, answering the research questions.

We prepared an overview of the selected studies based on a descriptive numerical summary and thematic analysis [15]: the summary provided a quantitative view of the articles’ main characteristics, while the analysis allowed a qualitative view of the articles and in-depth insights regarding the literature. That provided means to complement the descriptive numerical summary trends, contributing to fully answer our research questions.

## Results

Our *corpus* of works on LOS–P forecasting contains mostly journal papers (89%) authored by 125 authors who contributed to the topic with only one publication. Studies appeared in the proceedings of 3 conferences and 21 different journals, two of which with more than one publication: Journal of Perinatology (*n* = 2) and Journal of the American Academy of Child & Adolescent Psychiatry (*n* = 2). Research took place in nine different countries, four of which with more than one publication: USA (*n* = 17), Germany (*n* = 2), Brazil (*n* = 2), and Canada (*n* = 2). Studies belong to several knowledge areas (with prevalence in Pediatrics, *n* = 8, and Psychiatry, *n* = 4), medical specialties (e.g., cardiology and neurology), and medical departments (e.g., emergency and intensive care). Only one study is in the area of Computer Science, indicating that research on LOS–P prediction emphasizes healthcare applications rather than forecasting methods. The evolution of publications per year shows an increase in studies over the decades, beginning in the 1980s (*n* = 1) and with the most recent publications in 2020 (*n* = 2). In the decades of 1990 (*n* = 6), 2000 (*n* = 9), and 2010 (*n* = 10), the number of studies increased considerably, bending towards the use of Machine Learning modeling techniques.

In the thematic analysis, we divided studies according to three dimensions: the technical approach used to generate the forecasts, the medical department where the study took place, and the population analyzed. Table 2 summarizes our findings and serves as a guide to what has already been reported in the literature, addressing *RQ*_1_ and *RQ*_2_.

**Table 2:** Summary of studies regarding forecasting approach, and investigated department and population

Department	Population	Approach			# of articles
		*Regression*	*Machine Learning*	*Others*	
Emergency	Babies and children with bronchiolitis		[18]		2
	Pediatric trauma patients		[19]		
Neonatal Intensive Care units	Premature newborns	[20–22]			11
	Newborns	[23–26]		[27]	
	Chronically underweight newborns	[28–30]			
Pediatric Intensive Care units	Pediatric patients	[31]			1
Pediatric unit or hospital	Babies undergoing bidirectional Glenn procedure	[32]			3
	Children with hematological diseases complicated by febrile neutropenia	[33]			
	Pediatric patients with respiratory diseases		[2]		
Psychiatric unit or hospital	Children	[3, 34, 35]		[36]	6
	Teenagers	[3, 37, 38]		[36]	
	Young Adults	[38]			
Not specified	Premature newborns	[39]	[39]		5
	Newborns and babies undergoing cardiac surgery	[40]			
	Babies admitted for gastroenteritis	[41]			
	Pediatric patients		[42]		
	Pediatric victims of ATV accidents	[43]			
N° of articles		21	5	2	

The technical approaches were divided into Regression Analysis (used in 75% of the studies), Machine Learning techniques, and Others. Departments where studies took place were divided into six categories, one of which including articles that did not convey that information. The largest number of studies took place in Neonatal Intensive Care units (39.29%) and Psychiatric units or hospitals (21.43%), which are highly controlled areas with abundant LOS data. In those environments, regression analysis was the predominant forecasting technique. In opposition, LOS data from Emergency departments were exclusively modeled using Machine Learning techniques. Regarding the population analyzed, most studies used data from newborn patients (42.86%) or patients in specific situations (28.57%), e.g., victims of ATV accidents and children with hematologic malignancies complicated by febrile neutropenia. Fewer studies (32%) involved adolescent patients or young adults.

Table 3 characterizes the datasets used in the studies, addressing *RQ*_3_. The information in the table was collected through a critical appraisal of the *corpus* of articles, which was performed informally and without the aid of any specific tool.

**Table 3:** Characteristics of datasets used in the studies

Reference	Country	Sampling period	Sample size	Hospitals	LOS–P
[32]	USA	July 2001 to December 2007	100	1	Median: 20 days
[42]	USA	2016	5236	4200	Not informed
[29]	Not informed	January 1992 to December 1993	97	1	Mean: 52.8 days
[27]	USA	August 1999 to October 1999, and April 2002 to September 2002	908	1	Not informed
[38]	Not informed	Not informed	41	1	Mean: 18.02 months
[35]	USA	May 1988 to December 1989	96	1	Mean: 71.6 days
[21]	USA	July 2002 to December 2005	2254	Not informed	Not informed
[36]	Germany	Not informed	1001	13	Median: 104 days
[22]	Canada	Not informed	186	1	Not informed
[34]	Not informed	2010 to 2015	96	1	Mean: 18.56 days
[24]	USA	1990	558	1	Mean: 23 days
[41]	Australia	1995	514	58	Mean: 3.39 days
[26]	USA	2008 to 2011	23,551	125	Not informed
[3]	USA	1998 to 2001	1930	44	Mean: 10.4 days
[31]	USA	Not informed	2062	1	Mean: 3.5 days
[2]	China	January 2014 to April 2016	11,206	1	Not informed
[30]	Argentina, Chile, Paraguay, Peru and Uruguay	January 2001 to December 2008	7599	20	Not informed
[43]	USA	January 2000 to December 2009	420	Not informed	Not informed
[40]	Not informed	September 1993 to December 1997	458	1	Not informed
[33]	Brazil	February 2001 to May 2002	62	1	Mean: 10 days
[20]	USA	November 2014 to March 2017	152	14	Not informed
[25]	USA	October 1987 to July 1988	393	1	Mean: 23.8 days
[23]	South Africa	January 2007 to December 2008	3794	15	Mean: 17.9 days
[28]	USA	January 1994 to December 1996	314	2	Mean: 54.8 days
[37]	Canada	October 2005 to March 2010	2445	69	Mean: 16.31 days
[19]	USA	April 1994 to December 1997	7665	Not informed	Mean: 3.98 days
[18]	Ireland	1999	119	1	Not informed
[39]	Not informed	October 1989 to January 1996	2144	1	Not informed

Hospitalizations of pediatric patients were sampled in 13 countries; the USA (*n* = 14) is the country with the highest representation in the studies (50%). Data were collected between 1987 and 2017, covering from 9 to 120 months; data were mainly collected during the 1990s (*n* = 10) and 2000s (*n* = 10). Four studies do not specify the sampling period; the majority performed the sampling in a period equal to or greater than 1 year (*n* = 22). Sample sizes range from 41 to 23,551 observations. Most studies (*n* = 15) took place at a single location, indicating a low concentration of multicenter studies. Studies’ LOS–*P* values display averages or medians ranging from 3.39 days to 18.02 months, with more than 40% of the articles not reporting this information (*n* = 12). The longest LOS occurred in hospitals or Psychiatric units (ranging from 2 to 18 months). In Neonatal Intensive Care units, LOS–P averages vary from 23 to 54.8 days, with the highest averages (54.8 and 52.8 days) concentrated in the population of very low-weight neonates.

References: [32] Anderson et al. (2009) [42]; Balan et al. (2019) [29]; Bannwart et al. (1999) [27]; bender et al. (2012) [38]; Browning (1986) [35]; gold et al. (1993) [21]; Hintz et al. (2009) [36]; Höger et al. (2002) [22]; Jeremic and tan (2008) [34]; Kavanaugh et al. (2019) [24]; Khoshnood et al. (1996) [41]; lee et al. (2005) [26]; lee et al. (2016) [3]; Leon et al. (2006) [31]; Levin et al. (2012) [2]; ma et al. (2020) [30]; Marshall et al. (2012) [43]; Nagarsheth et al. (2011) [40]; Parkman and woods (2005) [33]; Pastura et al. (2004) [20]; Paul et al. (2020) [25]; Pearlman et al. (1992) [23]; Pepler et al. (2012) [28]; Rendina (1998) [37]; Stewart et al. (2013) [19]; Walczak and Scorpio (2000) [18]; Walsh et al. (2004) [39]; Zernikow et al. (1999)

Methods used to build the forecasting models are divided into three groups, according to the stage of model development they propose to address: (*i*) pre-processing, which prepares the data prior to modeling (see Table 4); (*ii*) variable selection, which optimizes the forecasting model by improving its precision and interpretability using the most informative variables (see Table 5); and (*iii*) cross-validation, which evaluates the performance of the model in different datasets (see Table 6). Several statistical and machine learning methods are proposed at each stage of model development, with references to works using each method listed in Tables 4, 5 and 6. In general, the reader does not have to search beyond those references to gather basic information on the methods. However, an overview of statistics-based methods is available in Kotz et al. [44] and machine learning-based methods in Bishop [45].

**Table 4:** Pre-processing modeling approaches

Pre-processing modeling approaches	Reference																												
	[32]	[42]	[29]	[27]	[38]	[35]	[21]	[36]	[22]	[34]	[24]	[41]	[26]	[3]	[31]	[2]	[30]	[43]	[40]	[33]	[20]	[25]	[23]	[28]	[37]	[19]	[18]	[39]	Frequency
Logarithmic transformation						✔		✔			✔							✔		✔	✔	✔	✔	✔	✔				10
Coding of categoric variables											✔			✔		✔			✔			✔		✔	✔	✔			8
Colinearity test	✔							✔		✔								✔					✔	✔	✔	✔			8
Missing data treatment				✔							✔				✔	✔								✔		✔			6
Variable categorization			✔																✔	✔									3
Feature engineering														✔		✔										✔			3
Data rescaling		✔																										✔	2
Outliers withdrawn										✔													✔						2

**Table 5:** Variable selection modeling approaches

Variable selection modeling approaches	Reference																												
	[32]	[42]	[29]	[27]	[38]	[35]	[21]	[36]	[22]	[34]	[24]	[41]	[26]	[3]	[31]	[2]	[30]	[43]	[40]	[33]	[20]	[25]	[23]	[28]	[37]	[19]	[18]	[39]	Frequency
Significance test				✔	✔		✔							✔					✔			✔							6
ANOVA			✔			✔		✔												✔			✔						5
Backward stepwise selection							✔						✔								✔								3
Forward stepwise			✔												✔													✔	3
Correlation analysis										✔											✔								2
Stepwise multiple cox regression																	✔												1
PCA																							✔						1

**Table 6:** Cross-validation modeling approaches and performances

Cross-validation modeling approaches and performances	Reference																												
	[32]	[42]	[29]	[27]	[38]	[35]	[21]	[36]	[22]	[34]	[24]	[41]	[26]	[3]	[31]	[2]	[30]	[43]	[40]	[33]	[20]	[25]	[23]	[28]	[37]	[19]	[18]	[39]	Frequency
Traditional Holdout		✔					✔	✔	✔					✔	✔	✔												✔	8
Temporal Holdout				✔									✔			✔							✔			✔			5
K-fold				✔																							✔		2
Performance (R^2^)	0.43	0.9415	0.63–0.82	0.66–0.79	0.36–0.43		0.38	0.097–0.237		0.242–0.278	0.66			0.22–0.30		0.694–0.831		0.329	0.04–0.225	0.47	0.4464	0.78	0.7027	0.51	0.1287				19
Performace (RMSE)													6.2–18.8			0,296 - 0,588													2
Performace (MAE)													4.2–14.6													2.5–4.26			2
Performance (Variance)						30.7–57%																							1
Performace (Average MSE)									0.08–0.38																				1
Performance (% Prediction up to 12 h)															27–46%														1
Performance (Correlation between forecasting models)																	0.92												1
Performance (% *Perfect Prediction)																										12.9–21.2%			1
Performance (% *Predictons up to one day)																										34–51.4%			1
Performance (Mean correct performance)																											60–80%		1
Performance (*Correlation between the predicted and actual LSPPH)																												0.85–0.92	1

The pre-processing methods reported in our *corpus* may be divided into (*i*) data cleaning methods to avoid modeling noise and (*ii*) methods to prepare and transform data to remove scale effects. The primary pre-processing method for data cleaning is the collinearity test, which evaluates the correlation level between independent variables. The test was reported in eight studies. To avoid noise in the model due to the excessive number of observations with missing data in the independent variables, six studies excluded incomplete observations from the datasets, and two adopted data imputation strategies. Two studies mention the withdrawal of outliers from the datasets before modeling.

The main pre-processing method for data transformation, adopted in ten studies, is the logarithmic transformation of LOS–*P* values to correct the positive asymmetric distribution of the dependent variable. The logarithmic transformation also reduces the effect of outliers, ensuring the residuals’ normality and stabilizing the variance.

Eight studies treat categorical variables as dummy variables or use specific codings that vary according to needs. Two studies use data rescaling through normalization and linear transformation. Other data preparation methods include the categorization of variables and feature engineering, which creates new variables by combining the ones available in the dataset.

Regarding the variable selection methods, most studies (*n* = 17) propose reducing the number of variables in the model to keep only the most informative. Variable selection aims to balance model simplicity and performance; however, it is noteworthy that most Machine Learning-based studies do not use any variable selection method (except for Zernikow et al. [39], who proposes the Forward Stepwise method). The most popular variable selection methods are the Analysis of Variance and the variable significance test.

Three studies use the stepwise backward method, which starts with all variables in the model and removes at each iteration the least significant one. Three studies use the stepwise forward method, starting with a model with no variables and adding the most significant one at each iteration. In addition to the methods mentioned above, others less predominant in the studies are correlation analysis, stepwise multiple Cox regression, and Principal Component Analysis (PCA).

Half of the studies in our *corpus* use cross-validation to validate model results, seeking its generalization. Cross-validation approaches are divided into three categories: traditional holdout, temporal holdout, and *k*-fold. The holdout method divides the dataset into two partitions (training and testing), which are mutually exclusive and vary according to the analyst’s preferences. Traditional holdout randomly splits the dataset, assuming that the frequency distributions do not change over time; temporal holdout divides the dataset taking into account the temporal evolution of the data. Except for two studies that do not mention the percentage of the dataset used in each partition [2, 30], all other studies use traditional holdout, varying the proportion of the dataset in the training and testing partitions as follows: 80–20% [31, 42], 75–25% [39], 70–30% [21], and 50–50% [3, 36]. The *k*-fold method randomly divides the dataset into *k* unique parts of the same size, trains the forecast model with *k* − 1 parts, and uses the remaining part for validation. The process is repeated *k* times, such that all parts are used in the validation step. Walsh et al. [18] use 5-fold, with the dataset divided into training, testing, and validation in five different ways, while Bender et al. [27] do not give details on the *k*-fold method used.

The performance of LOS–P forecasting models is measured using several metrics. The most used performance metric (*n* = 19) is the coefficient of determination (*R*^2^) that measures the proportion of the variability in the dependent variable captured by the model. In studies measuring model performance using *R*^2^, values ranged from 0.04 to 0.9415, with most studies reporting values greater than 0.5 (*n* = 8). Other performance metrics reported are the Root Mean Square Error (RSME) and the Mean Absolute Error (MAE). RMSE measures the standard deviation of model errors; studies that use this metric report values between 0.296 and 18.8 days [2, 26]. MAE measures the absolute average value of the differences between forecasts and actual observations; studies that used this metric reported values between 2.5 and 14.6 days [19, 26].

The independent variables (descriptors) used to build the forecasting models differ greatly between the studies since they analyze different populations and departments. Most studies use patient demographic descriptors such as age (neonatal age, postmenstrual age, gestational age, and chronological age), gender, and race. Studies modeling newborn populations usually include weight at birth as descriptor in the models. Other variables commonly reported are related to patients’ diagnoses, surgical procedures, and existing comorbidities. In addition, several studies use results from specific laboratory exams as descriptors in the prediction models (e.g., systolic blood pressure, respiratory rate, Apgar scores at 1 and 5 min, and neurocognitive presentation).

To start addressing *RQ*_5_, we list the main limitations and barriers reported in the LOS–P forecasting literature. They are: (i) lack of model generalizability due to differences across hospitals, (ii) lack of data on potentially useful LOS–P predictors, (iii) small sample sizes used to obtain forecasting models, and (iv) studies based on samples that do not adequately represent the entire population.

## Discussion

The analytical methods presented in Tables 4, 5 and 6 are not directly comparable since they are applied to different population samples, using various combinations of pre-processing, variable selection, and cross-validation approaches. However, our scoping review should help readers to identify which analytical pathways have been tested for specific populations and how they perform in terms of prediction. To identify works covering a certain population of interest and forecasting approach, the reader is directed to Table 2, with details mapped in Tables 4, 5 and 6.

The extensive utilization of regression analysis in LOS–P forecasting is aligned with findings in Hussain and Dunn [11], who reviewed studies that predicted LOS of thermal burned patients and reported that all forecasting models were based on multivariate regression analysis. We also reported a trend towards artificial intelligence-based forecasting models in recent years, which was not observed in previous review studies, e.g., Seaton et al. [14] reported only one model based on Artificial Neural Networks, while Almashrafi et al. [8] and Lu et al. [12] reported a model based on decision trees. The dependent variable used in all studies we reviewed was the continuous LOS; in opposition, Almashrafi et al. [8], Atashi et al. [10], Lu et al. [12], and Seaton et al. [14] presented studies that modeled the discrete LOS of patients using multivariate logistic regression.

In terms of the main pre-processing methods, regardless of the frequently observed positive asymmetric distribution of the LOS variable, only 35.7% of our reviewed studies used the logarithmic transformation, similar to what was reported by Lu et al. [12], in which 32% of studies used log-transformed LOS as the dependent variable. Most studies in our review did not mention the handling of missing data, a gap that was also reported by Atashi et al. [10].

Concerning the approaches for variable selection, reviews by Hussain and Dunn [11] and Peres et al. [9] reported that most studies selected variables to be included in the forecasting models through univariate analysis, while Seaton et al. [14] reported results similar to ours (i.e., analysis of variance and significance tests). Other authors did not report some variable selection techniques identified in our review (i.e., stepwise multiple Cox regression, correlation analysis, and Principal Components Analysis).

For cross-validation, previous reviews reported similar results to ours: Seaton et al. [14] concluded that over 50% of the authors investigated had validated results by splitting the sample, while Verburg et al. [13] reported that over 38% of the studies used simple random sample split. Bootstrapping cross-validation, an approach not identified in our studies, was mentioned by Atashi et al. [10] and Verburg et al. [13].

Regarding the performance of LOS–P forecasting models, the predominance of the coefficient of determination (*R*^2^) was also found in the studies reported by Seaton et al. [14] and Hussain and Dunn [11], with values ranging from 0.158 to 0.75. In opposition, reviews by Atashi et al. [10] and Verburg et al. [13] found that Pearson’s correlation coefficient was the preferred performance metric.

Most studies report benefits from using LOS–P forecasts for the hospital ecosystem. We identified five dimensions positively affected by the use of forecasting models: (*i*) patient care, (*ii*) costs, (*iii*) hospital management, (*iv*) quality measurement, and (*v*) updating of medical practices. The two main benefits reported in the patient care dimension are providing families information about the expected discharge date and preventing complications associated with prolonged hospitalizations. Hintz et al. [21] suggested that LOS–P prediction allows a better understanding of risk factors associated with prolonged stays. Identifying such patients may direct hospitals towards more aggressive treatments and the provision of specialized care to prevent complications [34, 41].

Benefits associated with the cost dimension are estimates of financial values spent on hospitalization and cost reduction for the hospital. LOS–P forecast contributes to the hospital’s strategic planning and guides medical care, reducing the length of the patient’s stay and, consequently, hospitalization costs [29, 30]. Hospital management can bring several benefits to the hospital, being directly related to the other dimensions. Studies report management areas positively affected by LOS–P forecasts, such as resource allocation and planning [23, 26], patient flow management [19, 31], hospital bed management [2, 41], optimization of decision making [18, 22], and shift staff scheduling [27, 39]. As for the benefits of measuring patient care quality, studies advocate the monitoring of hospital performance and the standardization of care across hospitals. Hospital performance may be assessed by measuring the effect of hospital-related variables in the LOS–P model [41] or by using predicted LOS values as reference [23], and the difference between the expected and actual LOS values as a service quality indicator [39]. The last dimension of benefits is related to the detection of variations in historical patterns due to changes in medical practices resulting from updating LOS–P models. Walczak and Scorpio [19] report that the use of neural network models makes solutions non-static; as medical practices evolve, the prediction model is quickly adapted through continuous learning based on new datasets.

Works included in this scoping review present some gaps related to the studied population and departments. Most LOS–P forecasting studies are focused on newborn patients; the analysis of child and adolescent patients constitutes a research opportunity. Studies are predominantly carried out at Neonatal Intensive Care units and hospitals or Psychiatric units. There is an opportunity to develop studies in Emergency departments and Pediatric Intensive Care units. Datasets including all pediatric ages and departments of pediatric hospitals are also needed to compare the performance between general and dedicated forecasting models. We reported a small number of multicenter studies and therefore identified such studies as a research opportunity. By doing it so, it would be possible to compare how the LOS-P forecasting would be impacted, for example, by patients from different locations and submitted to different treatments.

## Conclusions

Our study aimed to identify works devoted to LOS–P forecasting through a scoping review of the literature. LOS forecasting is a tool to improve the management of resources in healthcare environments, helping organizations to cope with high demands and quality requirements. Although some literature review papers investigate the prediction of LOS in different environments and patient populations, we bridge a gap in the literature regarding reviews focusing on pediatric patients, a complex population with a high mortality risk that represents a challenge for hospital managers. Studies from our *corpus* rarely report the proposed forecasting models’ real applicability, mostly indicating only qualitative benefits. The development of studies reporting the use of LOS–P forecasting models as tools for resource optimization through the qualitative and quantitative reporting of benefits remains a promising research direction.

This review article also has practical implications as it provides arguments to evaluate the applicability of different modeling techniques in forecasting the LOS–P in different environments and types of pediatric populations. That allows informing family members about the patient’s expected discharge date, which is particularly critical for pediatric patients. Additionally, hospital resources’ allocation and planning can be significantly improved by properly estimating the LOS–P. More assertive and precise LOS–P forecast models can significantly avoid waste of labor and materials shortages in hospitals, reducing costs and increasing efficiency.

Our study has some limitations. First, to ensure repeatability and allow a detailed analysis of the forecasting approaches reported in the literature, we limited our search to English databases. Second, performance comparisons were constrained by the studies’ heterogeneity (e.g., different sample sizes, locations, methods, and performance metrics). Third, we do not analyze classification studies, which are concerned with classes of LOS instead of continuous forecasts. Fourth, CINAHL and Embase were not included in the search for studies as they cover essentially the same titles as Medline and Scopus, which could artificially inflate the initial count of articles. However, those databases provide unique indexing and citations that could have led to studies not retrieved in the four databases selected. Fifth, authors of articles in our *corpus* were not contacted to identify further related studies not discovered in the database searches. Finally, since the age range of pediatric patients varies in the literature, we included all studies that defined their population as pediatric, ranging from newborns to young adults.

## Supplementary Information

**Additional file 1.**

**Additional file 2.**

**Additional file 3.**

## Data Availability

All data generated or analyzed during this study are included in this published article.
