# Supplementary material for: Forecasting the length-of-stay of pediatric patients in hospitals: a scoping review
Source: BMC Health Serv Res. 2021 Sep 8;21:938. doi: 10.1186/s12913-021-06912-4 (PMC8428133; doi:10.1186/s12913-021-06912-4)
Supplement: Supplementary file 1 — Additional file 1. [file 12913_2021_6912_MOESM1_ESM.docx]

**Supplementary Material 1**

| **Database** | **Forecast models** | **Length of stay in hospital** | **Pediatric patients** | **File type** | **Language** |
| --- | --- | --- | --- | --- | --- |
| Medline | **Title, Abstract:** Predict* OR/AND Model OR/AND Prognos* OR Forecast* OR/AND Regression OR/AND Estimat* | **Title:** "Length of Stay" OR/AND "Hospital Days" OR/AND "Length of Hospital Stay" OR/AND "Duration of Stay" OR/AND "Patient Stay" | **Title:** child* OR/AND pediatric OR/AND paediatric OR/AND kid OR/AND youth* OR/AND adolescen* OR/AND neonat* OR/AND newborn* OR/AND infant* | Academic journals | English |
| Science Direct | **Title, Abstract, Keywords:** Predict OR/AND Model OR/AND Prognosis OR/AND Prognostic OR/AND Forecast OR/AND Regression OR/AND Estimate | **Title:** "Length of Stay" OR/AND "Hospital Days" OR/AND "Length of Hospital Stay" OR/AND "Duration of Stay" OR/AND "Patient Stay" | **Title:** children OR/AND pediatric OR/AND paediatric OR/AND kid OR/AND youth OR/AND adolescent OR/AND neonate OR/AND newborn OR/AND infant | Review Articles  Research Articles | - |
| Scopus (Elsevier) | **Article Title, Abstract, Keywords:** Predict* OR/AND Model OR/AND Prognos* OR/AND Forecast* OR/AND Regression OR/AND Estimat* | **Article Title:** "Length of Stay" OR/AND "Hospital Days" OR/AND "Length of Hospital Stay" OR/AND "Duration of Stay" OR/AND "Patient Stay" | **Title:** child* OR/AND pediatric OR/AND paediatric OR/AND kid OR/AND youth* OR/AND adolescen* OR/AND neonat* OR/AND newborn* OR/AND infant* | Article  Conference Paper  Review | English |
| Web of Science | **Title, Abstract, Keywords:** Predict* OR/AND Model OR/AND Prognos* OR/AND Forecast* OR/AND Regression OR/AND Estimat* | **Title:** "Length of Stay" OR/AND "Hospital Days" OR/AND "Length of Hospital Stay" OR/AND "Duration of Stay" OR/AND "Patient Stay" | **Title:** child* OR/AND pediatric OR/AND paediatric OR/AND kid OR/AND youth* OR/AND adolescen* OR/AND neonat* OR/AND newborn* OR/AND infant* | Article  Proceedings Paper  Review | English |
