# Supplementary material for: Forecasting the length-of-stay of pediatric patients in hospitals: a scoping review
Source: BMC Health Serv Res. 2021 Sep 8;21:938. doi: 10.1186/s12913-021-06912-4 (PMC8428133; doi:10.1186/s12913-021-06912-4)
Supplement: Supplementary file 2 — Additional file 2. [file 12913_2021_6912_MOESM2_ESM.docx]

**Supplementary Material 2**

| **Paper** | **Title** | **DOI** | **Exclusion criteria** |
| --- | --- | --- | --- |
| 1 | A validated clinical model to predict the need for admission and length of stay in children with acute bronchiolitis | 10.1097/00063110-200410000-00005 | Forecasting models are for classification and not for LOS prediction. |
| 2 | Ability of the pediatric comprehensive severity index to predict length of stay and costs | 10.1097/00003246-199901001-00011 | Study focuses on individual predictors of LOS. |
| 3 | Estimating neonatal length of stay for babies born very preterm | 10.1136/archdischild-2017-314405 | The study does not present LOS modeling. It estimates the length of stay through calculation of averages. |
| 4 | Estimating the neonatal length of stay for preterm babies in a saudi tertiary hospital | 10.4103/jcn.JCN_115_19 | The study does not present LOS modeling. It calculates the median length of stay for each week of gestation at birth. |
| 5 | Excess body weight in children may increase the length of hospital stay | [http://dx.doi.org/10.6061/clinics/2015(02)03](http://dx.doi.org/10.6061/clinics/2015(02)03 ) | The study does not present LOS modeling. It compares the length of stay between different groups (overweight and non-overweight children). |
| 6 | Factors predicting length of stay in an adolescent psychiatric unit: A retrospective study [Durée d’hospitalisation en pédopsychiatrie : étude rétrospective des facteurs prédictifs sur deux ans en unité d’adolescents] | 10.1016/j.neurenf.2020.03.004 | Study in French. |
| 7 | Length of stay, conditional length of stay, and prolonged stay in pediatric asthma | [10.1111/1475-6773.00150](https://doi.org/10.1016/j.jpedsurg.2016.05.006) | The study does not present LOS modeling. It compares the length of stay between different groups (hospitalizations in New York and in Pennsylvania). |
| 8 | Machine Learning-based Prediction of Prolonged Length of Stay in Newborns | 10.1109/ICMLA.2018.00236 | The forecasting models are for classification and not for prediction. |
| 9 | Negotiating managed care contracts: Do historical and demographic risk factors predict neonatal length of stay (LOS) | <https://doi.org/10.1016/S0002-9378(97)80489-9> | Just a summary, not bringing enough information to be included in the review. |
| 10 | Pediatric rehabilitation: trends in length of stay | [10.3233/PRM-130232](https://doi.org/10.1016/j.ppedcard.2010.11.011) | The study does not present LOS modeling. It compares the length of stay between different groups (diseases). |
| 11 | Predicting duration of stay in a pediatric intensive care unit: A Markovian approach | 10.1016/S0377-2217(99)00296-9 | The study does not present LOS modeling. It uses Markov Chains to estimate the time a patient spends on a given severity scale (high, low, and medium). |
| 12 | Predicting length of hospital stay in a pediatric ward | - | The forecasting models are for classification and not for prediction. |
| 13 | Predicting mortality, hospital length of stay and need for surgery in pediatric trauma patients | [10.1016/j.cjtee.2017.04.011](https://doi.org/10.1016/j.ppedcard.2010.11.011) | The forecasting models are for classification and not for prediction. |
| 14 | Predicting the length of stay using Bayesian model selection for Neonatal heart rate monitoring | 10.2316/P.2011.718-104 | Access to the material not available. |
| 15 | Prediction of Length of Hospital Stay in Preterm Infants a Case-Based Reasoning View | 10.1007/978-3-319-39630-9_10 | The study does not present LOS modeling. It uses a formal framework based on Logic Programming for Knowledge Representation and Reasoning. |
| 16 | Predictors of hospital length of stay in pediatric Henoch-Schonlein purpura | 10.1007/s00296-015-3257-6 | The forecasting models are for classification and not for prediction. |
| 17 | Predictors of length of hospital stay among preterm infants admitted to neonatal intensive care unit: Data from a multicentre collaborative network from India (INNC: Indian National Neonatal Collaborative) | 10.1111/jpc.15031 | The study does not present LOS modeling. It calculates the median length of stay for each week of gestation at birth. |
| 18 | Provider-level effects on psychiatric inpatient length of stay for youth with mental health and substance abuse disorders | 10.1097/MLR.0b013e318158aee7 | The study does not present LOS modeling. It compares the length of stay between different groups (patient and installation factors). |
