# Supplementary material for: Forecasting the length-of-stay of pediatric patients in hospitals: a scoping review
Source: BMC Health Serv Res. 2021 Sep 8;21:938. doi: 10.1186/s12913-021-06912-4 (PMC8428133; doi:10.1186/s12913-021-06912-4)
Supplement: Supplementary file 3 — Additional file 3. [file 12913_2021_6912_MOESM3_ESM.docx]

**Supplementary Material 3**

**Data charting process**

| Title | Pre-processing modeling approaches |
| --- | --- |
| Authors | Variable selection modeling approaches |
| Type of paper (journal or conference) | Cross-validation modeling approaches |
| Journal name or Conference name | Performances |
| Publication year | Variables |
| First author country | Significant variables |
| Knowledge area | Department |
| Country of the data sampling | Population |
| Data sampling period | Approach |
| Sample size | Managerial implications |
| Patient ages | Barriers and limitations |
| Number of hospitals | Opportunities for future research |
| LOS-P mean or median |  |
